# Selenium and Thyroid Disease: From Pathophysiology to Treatment

**DOI:** 10.1155/2017/1297658

**Published:** 2017-01-31

**Authors:** Mara Ventura, Miguel Melo, Francisco Carrilho

**Affiliations:** ^1^Department of Endocrinology, Diabetes and Metabolism, Centro Hospitalar e Universitário de Coimbra, Coimbra, Portugal; ^2^Faculty of Medicine, University of Coimbra, Coimbra, Portugal

## Abstract

*Introduction.* Selenium is a micronutrient embedded in several proteins. In adults, the thyroid is the organ with the highest amount of selenium per gram of tissue. Selenium levels in the body depend on the characteristics of the population and its diet, geographic area, and soil composition. In the thyroid, selenium is required for the antioxidant function and for the metabolism of thyroid hormones. *Methods.* We performed a review of the literature on selenium's role in thyroid function using PubMed/MEDLINE. *Results.* Regarding thyroid pathology, selenium intake has been particularly associated with autoimmune disorders. The literature suggests that selenium supplementation of patients with autoimmune thyroiditis is associated with a reduction in antithyroperoxidase antibody levels, improved thyroid ultrasound features, and improved quality of life. Selenium supplementation in Graves' orbitopathy is associated with an improvement of quality of life and eye involvement, as well as delayed progression of ocular disorders. The organic form of selenium seems to be the preferable formulation for supplementation or treatment. *Conclusion.* Maintaining a physiological concentration of selenium is a prerequisite to prevent thyroid disease and preserve overall health. Supplementation with the organic form is more effective, and patients with autoimmune thyroiditis seem to have benefits in immunological mechanisms. Selenium supplementation proved to be clinically beneficial in patients with mild to moderate Graves' orbitopathy.

## 1. Introduction

Selenium is a micronutrient first described in 1817; its name derives from the Greek “σελήνη—Selene” meaning moon, referring to the bright and grey appearance of this compound when it is melted [1]. Selenium levels in the body are dependent on the population's characteristics and its diet and geographical area (mainly on the soil composition) [1, 2]. This micronutrient has been studied over the last years, and scientific reports have revealed its crucial role in the maintenance of immune-endocrine function, metabolism, and cellular homeostasis. The thyroid gland is characterized by a high concentration of selenium, which is incorporated into selenoproteins. Some of these selenoproteins have an important antioxidant activity, contributing to the antioxidant defense in the thyroid by removing oxygen free radicals generated during the production of thyroid hormones. Being incorporated into iodothyronine deiodinases, selenium plays also an essential role in the metabolism of thyroid hormones.

### 1.1. Requirements and Natural Sources of Selenium

Selenium can be available both in organic compounds (selenomethionine and selenocysteine) and in inorganic compounds (selenite and selenate) [1]. Considering that the organic form has better absorption, it seems to be the preferable formulation for supplementation or treatment [3]. Selenomethionine is found in vegetable sources (especially cereals), selenium yeast, and other selenium supplements [1]. Selenium is incorporated into body proteins in place of methionine; therefore, supplements containing selenomethionine are those which have more bioavailable selenium. In turn, selenocysteine, a selenium analogue of the amino acid cysteine, is found mainly in animal foods. The inorganic forms (selenate and selenite) are the components of dietary supplements. According to a study performed in Belgium, the main sources of selenium are meat products (31%), followed by fish (19%), pasta or rice (12%), and bread or cereals (11%) [4]. Most of the selenium is absorbed in the small intestine (50–80%) and excreted by the kidneys (60%); intestinal excretion of selenium is about 35% and only 5% is excreted in sweat or saliva [1]. By mechanisms not yet completely clarified, reduced selenium levels are found in smokers and with advanced age; selenium depletion has also been associated with the consumption of eggs, white rice, alcohol, and coffee [5]. The daily intake of selenium is variable according to the geographical area, as mentioned previously (Table 1). Indeed, in Europe, dietary selenium intake is about 40 *μ*g per day, and in the USA, it was reported to be 93 *μ*g per day in women and 134 *μ*g per day in men [2]. Table 2 shows the recommended daily allowances of selenium for adults [6, 7]. In general, no difference exists in the recommended dietary allowance of selenium between men and women [8].

Even though the daily intake of selenium in Europe does not reach the recommended levels, the opposite spectrum—excess of selenium in the body with toxic effects—can also occur in rare occasions. This situation, known as selenosis, generally arises when this micronutrient's concentrations exceed 400 *μ*g per day [8]. This rare situation was mainly reported by epidemiological studies in populations living in areas with high selenium concentration in the soil and can result from acute poisoning or prolonged exposure to high levels of selenium [9]. Selenium toxicity symptoms include nausea, vomiting, abdominal pain, diarrhea, hair loss, brittle nails, peripheral neuropathy, and the characteristic smell of garlic in sweat and breath. MacFarquhar et al. [10] reported 201 cases of acute selenium toxicity associated with a misformulated supplement. The implicated product was marketed as a dietary supplement that contains multiple nutrients and 200 *μ*g per fluid ounce of sodium selenite (30 mL). Among the 156 patients with available data, the median estimated amount of selenium ingested was 41585 *μ*g/day, with a range of 3400–244800 *μ*g/day. After the supplement was suspended, serum and urine selenium concentrations decreased gradually with time and returned to normal by weeks 1 to 2 for urine and started to normalize at week 6 for serum.

The level of selenium in the plasma depends directly on the selenium intake and correlates well with the organic availability of this nutrient.

## 2. Methods

**Figure 1:**
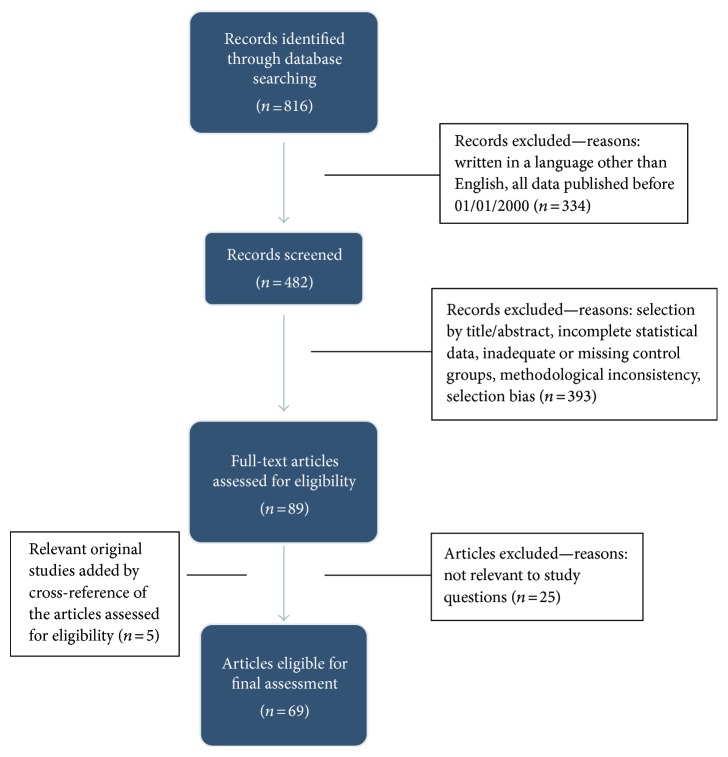
Flowchart of the selection process.

We performed a review of the literature on selenium's role in thyroid function using the PubMed/MEDLINE database, including the terms “selenium” and “thyroid.” A total of 816 articles were identified up to September 2016. Of these, we selected the articles published after January 2000 and excluded articles written in a non-English language, not relevant to the present review, with inconsistent methodology, or with evident selection bias (Figure 1). In order to include original studies, 5 publications were added by cross-reference. At the end, we selected 69 publications for final assessment.

## 3. Results and Discussion

### 3.1. Selenium Homeostasis and the Thyroid Gland

The vital role of selenium in thyroid function began to be questioned because of a condition described in Zaire (Democratic Republic of the Congo), known as myxedematous endemic cretinism, which was characterized by a deficit of iodine and selenium, hypothyroidism, myxedema, developmental problems, and intellectual disability [11]. From that moment, more studies were conducted to investigate the role of this nutrient in the thyroid. In fact, it was found that selenium deficiency decreases the synthesis of thyroid hormones, as it decreases the function of selenoproteins, in particular iodothyronine deiodinases (DIOs), which are responsible for the conversion of T4 to T3. This decreased production of thyroid hormones leads to the stimulation of the hypothalamic-pituitary axis due to the lack of negative feedback control, increasing TSH production. TSH stimulates the DIOs to convert T4 to T3 [12], with consequent production of hydrogen peroxide, which is not adequately removed by less active glutathione peroxidases (GPx) and accumulates itself in the thyroid tissue causing thyrocyte damage with subsequent fibrosis.

The thyroid gland is characterized by a high tissue concentration of selenium (0.2–2 *μ*g/g), being the organ with the highest amount of selenium per gram of tissue, because it contains most of the selenoproteins [1, 13]. Since it is incorporated into selenoproteins, which have an important antioxidant activity, selenium contributes to the antioxidant defense in the thyroid, by removing oxygen free radicals generated during the production of thyroid hormones [14, 15]. Being incorporated into iodothyronine deiodinases, selenium plays also an essential role in the metabolism of thyroid hormones [1, 16].

So far, about 25 selenoproteins were described [17]. Table 3 depicts selenoproteins which play a major role in thyroid homeostasis. The iodothyronine deiodinases control the thyroid hormone turnover and catalyze the conversion of T4 to its biologically active form, T3, through the removal of an iodine atom from the external ring [18]. They can also inactivate thyroid hormones by the removal of an iodine atom of the inner ring, with the conversion of T4 to reverse T3 (rT3), the inactive metabolite. Glutathione peroxidases are responsible for glandular protection, since they remove the excess of oxygen free radicals produced during normal synthesis of the thyroid hormones [19, 20].

Selenoprotein P is the main source of selenium in plasma; therefore, it constitutes the main transporter and distributor of this micronutrient [21]. It is produced by hepatocytes and has a crucial role in selenium homeostasis, since it ensures selenium retention in the body and promotes its distribution to the liver and extrahepatic tissues, including its transportation to the brain in conditions associated with nutrient deficit [22]. However, it seems that in the case of selenium deprivation and in the absence of this transporter, endocrine organs and the brain are preferentially supplied. The thyroid gland may be able to accumulate, retain, and recycle selenium efficiently, even in the absence of selenoprotein P [23].

### 3.2. Selenium in Thyroid Pathology

#### 3.2.1. Autoimmune Thyroiditis

Several studies have focused on the importance of selenium in thyroid function and autoimmune processes, aiming at understanding if supplementation of this micronutrient may have an impact on the evolution of thyroid disease. In fact, the effect of selenium supplementation on the evolution of Hashimoto's thyroiditis, a condition characterized by the presence of antithyroperoxidase and antithyroglobulin antibodies (TPOAb and TgAb, resp.), has been addressed in several publications. Gartner et al. [24] conducted a study that evaluated the effect of supplementing diet with 200 *μ*g sodium selenite per day during 90 days on the level of TPOAb and TgAb in patients with autoimmune thyroiditis; 71 patients with autoimmune thyroiditis under therapy with levothyroxine and with high levels of TPOAb and/or TgAb were evaluated. Patients were divided into two groups: one group that was supplemented with sodium selenite and the other group that just kept therapy with levothyroxine. At the end of the study, the concentration of TPOAb decreased by 40% in the group treated with selenium (versus 10% in the placebo group) and in 9 of 36 patients (25%), TPOAb completely normalized; during this period, thyroid echogenicity also improved. In this trial, patients receiving selenium supplementation reported better well-being compared with the placebo group.

On the other hand, Duntas et al. [25] conducted a study including 65 patients with autoimmune thyroiditis, aged between 22 and 61 years old, that had been under treatment with levothyroxine and were divided into two groups: one group received 200 *μ*g selenomethionine per day and the other received placebo. The aim of this study was to assess the effect of the treatment with selenium in patients with autoimmune thyroiditis through the impact on the level of TPOAb and TgAb after 3 and 6 months. In the group supplemented with selenomethionine, the level of TPOAb decreased by 46% at 3 months and 55.5% at 6 months, compared to a decrease of only 21% and 27%, respectively, at 3 and 6 months, in the group under isolated therapy with thyroxine. Nevertheless, there was no statistically significant difference in the level of TPOAb or in the concentration of TSH, free T4, and T3 between the two groups [25].

**Figure 2:**
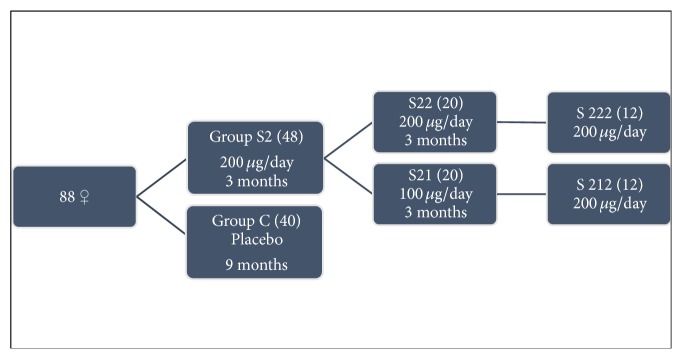
Adapted from [26].

Turker et al. [26] evaluated the effects of long-term (9 months) supplementation with variable doses of selenomethionine (100/200 *μ*g per day) on autoimmune thyroiditis, particularly on the concentration of TPOAb and TgAb. In their study, 88 women with autoimmune thyroiditis under therapy with thyroxine were included, who were allocated to two groups according to their initial level of serum TSH and TPOAb and age (Figure 2). The authors concluded that replacement with selenomethionine suppresses serum concentrations of TPOAb, but the suppression required doses greater than 100 *μ*g/day to maximize glutathione peroxidase activity. Furthermore, they also found that the suppression rate decreases with time. In fact, the group supplemented during the 9 months with 200 *μ*g/day selenomethionine had a sharp decrease in serum levels of TPOAb until 6 months of treatment, after which the values tended to level off (26.6% at 3 months, 26.2% at 6 months, and 3.6% at 9 months). In contrast, the group of patients supplemented in the second quarter of the study with 100 *μ*g/day showed an increase of 38.1% in the level of TPOAb. However, when this same group of patients received again supplementation with 200 *μ*g/day, there was a decrease of 30.3% in the level of TPOAb. Thereby, the authors demonstrated that the oral administration of 200 *μ*g/day of selenomethionine reduces effectively serum levels of TPOAb and even patients with selenium intake above the recommended levels may benefit from treatment with this dose.

In another study, Gartner and Gasnier [27] demonstrated on a prospective placebo-controlled trial performed in 47 patients with autoimmune thyroiditis treated with levothyroxine that supplementation with 200 *μ*g/day of sodium selenite for 6 months significantly reduces the concentrations of TPOAb in patients who already were under selenium supplementation or started to receive selenium after placebo; on the other hand, in patients who discontinued supplementation, a subsequent increase in TPOAb was found.

A prospective study by Nacamulli et al. revealed that supplementation with physiological doses of selenium (80 *μ*g/day of sodium selenite) for 12 months reduces the echogenicity of the thyroid and TPOAb and TgAb levels, without affecting significantly the concentration of TSH or T4 [28].

Esposito D. et al. studied the effect of 6 months' supplementation with 166 *μ*g/day selenomethionine on the thyroid function (evaluated through the level of TSH, thyroid hormones, thyroid peroxidase antibodies, thyroglobulin antibodies, and thyroid echogenicity) in untreated euthyroid patients with Hashimoto's thyroiditis. The authors also measure CXCL10 levels to evaluate the possibility of a modulation of the autoimmune mechanism by selenomethionine. The authors conclude that TSH, the levels of thyroid hormones and TPOAb, thyroid echogenicity, and CXCL10 concentration did not show a statistical difference at baseline and after 3 and 6 months between the control and the supplemented group. In fact, they observed an increase in FT3 levels after 3 and 6 months and a decrease in FT4 levels after 3 months in the group supplemented with selenium versus baseline levels; in the control group, the authors observed a decrease in FT3 after 3 and 6 months when compared to baseline.

In pregnancy, supplementation of selenium appears to influence thyroid function and may be beneficial. Mao et al. [29] evaluated the effect of supplementation between 12 and 14 weeks of gestation with 60 *μ*g/day selenium versus placebo in women with mild to moderate iodine deficiency. They found that the group supplemented with selenium did not show a significant decrease in thyroid peroxidase antibodies, though a minor change of thyroid function without clear clinical meaning occurred. Negro et al. [30] recruited 2143 pregnant women with autoimmune thyroiditis in euthyroidism to evaluate the effect of selenium supplementation, during and after pregnancy. Of the 2143 women selected, 169 were positive for thyroid peroxidase antibodies (TPOAb+) and were randomly divided into two groups: 77 pregnant women received 200 *μ*g/day selenomethionine and 74 received placebo. The authors found that in the group supplemented with 200 *μ*g/day selenomethionine during pregnancy and postpartum a decrease in the progression of autoimmune thyroiditis was observed; in fact, they found a reduction of TPOAb levels, improved thyroid echogenicity, decreased incidence of thyroid dysfunction in the postpartum period, and decreased permanent hypothyroidism [30].

It is important to note that, in most of the studies that focus on the relevance of selenium to thyroid disease, the authors did not measure selenium concentration prior to, during, and after supplementation. Furthermore, the most frequent primary outcome measurement was thyroid Ab levels, so at the present time, there is no recommendation for selenium supplementation in patients with autoimmune thyroiditis.

Recently, some clinical trials were designed to answer some of these still open questions. The CATALYST trial (“The chronic autoimmune thyroiditis quality of life selenium trial”) is an ongoing randomized controlled trial that enrolled 472 patients with autoimmune thyroiditis treated with levothyroxine (LT4). Their primary objective is to investigate the effect of 12 months' 200 *μ*g selenium-enriched yeast supplementation versus placebo on thyroid-related quality of life. Secondary objectives are to evaluate the effect of selenium supplementation versus placebo on LT4 dosage, serum T3/T4 ratio, serum TPOAb concentration, plasma selenium concentration, and immunological and oxidative stress biomarkers. Unlike some other studies about this issue, in this trial, plasma selenium concentrations will be measured periodically to assess selenium intake. This is also the first study that will evaluate selenium's mechanisms of action in autoimmune thyroiditis and the effect of selenium supplementation on LT4 dosage. According to the study protocol, this trial is scheduled to finish in 2018 [31].

#### 3.2.2. Selenium, Thyroid Volume, and Thyroid Nodules

Other studies have also evaluated the relationship between thyroid volume and selenium concentration [32–34]. Many of them were small studies and operator dependent but seem to suggest that there is an inverse relationship between the concentration of selenium in the plasma or urine (selenuria) and the thyroid volume or its hypoechogenicity. Rasmussen et al. [32] conducted a cross-sectional study in Denmark to evaluate the association between serum selenium concentration and thyroid volume, as well as between serum selenium concentration and risk for an enlarged thyroid in an area with iodine deficiency before and after iodine supplementation was initiated. The authors concluded that low serum selenium concentration was associated with a higher risk for an enlarged thyroid gland and for the development of thyroid nodules.

Regarding the sample size, one of the most impressive studies in this area was conducted by Wu et al. [34]. The authors selected 6152 patients by stratified cluster sampling: 3038 were defined as adequate-selenium county participants and 3114 were defined as low-selenium county, with a median difference in the selenium concentration between the groups of almost twofold. They aimed at investigating whether the prevalence of thyroid disease differed in two areas of China with different soil/crop selenium concentrations. The authors concluded that the prevalence of thyroid diseases (hypothyroidism, subclinical hypothyroidism, autoimmune thyroiditis, and an enlarged thyroid) was significantly lower in the adequate-selenium county than in the low-selenium county.

Most of these studies seem to demonstrate that selenium deficiency is associated with higher prevalence of thyroid disease, but further data are needed to assess if selenium can be protective against multinodular goitre and autoimmune thyroiditis.

#### 3.2.3. Selenium and Graves' Disease

Several groups have analyzed the importance of selenium supplementation in patients with Graves' disease. Vrca et al. [35] evaluated the effect of supplementation with a fixed combination of antioxidants (vitamins C and E, beta-carotene, and selenium) on the speed of attaining euthyroidism in a group of patients with Graves' disease treated with methimazole. The results of this study indicated that patients who received supplementation with antioxidants in addition to therapy with methimazole attained euthyroidism faster than the group treated with methimazole only. Another study by Wang et al. enrolled 41 patients with recurrent Graves' disease who were under treatment with methimazole [36]. The aim of this study was to evaluate the efficacy of selenium therapy on recurrent hyperthyroidism caused by Graves' disease. Twenty-one patients were supplemented with selenium in addition to methimazole for 6 months. The authors found that both FT4 and FT3 decreased more in the selenium group than in the control group at 2 months; they also found that the TSH level increased more and the TRAb level was significantly lower in the first group of patients. In fact, the proportion of patients with normal TRAb level at the final follow-up visit was also significantly higher in the selenium group. This study suggests that antioxidants administered together with antithyroid drugs may lead to a faster control of clinical manifestations and a faster normalization of thyroid function.

Graves' orbitopathy is a condition with a close clinical relationship with hyperthyroidism, which is understandable given that both have a common etiological basis. In fact, nearly half of the patients with Graves' disease have symptoms of Graves' orbitopathy [37]. In this regard, the importance of selenium supplementation in patients with Graves' orbitopathy has been under investigation. Marcocci et al. [38] carried out a randomized, double-blind, placebo-controlled trial to determine the effect of selenium or pentoxifylline in 152 patients with mild Graves' orbitopathy. The patients were given sodium selenite 100 *μ*g twice daily, pentoxifylline 600 mg twice daily, or placebo for 6 months; after that, the patients were followed up for 6 more months after treatment had been withdrawn. They found that treatment with selenium, but not with pentoxifylline, was associated with improved quality of life, less eye involvement, and delayed progression of Graves' orbitopathy at 6 months. The patients were subsequently reassessed at 12 months (after 6 months without selenium, pentoxifylline, or placebo supplementation), and the results obtained in the first assessment were confirmed. Although the evidence concerning selenium benefits in Graves' orbitopathy comes from this single randomized controlled study, a recommendation for its use in mild cases was incorporated into the recent guidelines from the European Group On Graves' Orbitopathy (EUGOGO) [39].

The ongoing GRASS trial (GRAves' disease Selenium Supplementation trial) enrolled 492 patients with Graves' hyperthyroidism, treated with antithyroid drugs, which were randomized to intervention with 200 *μ*g/day of selenium-enriched yeast versus placebo for 24 to 30 months. The purpose of this trial is to investigate if selenium addition to antithyroid drugs will lead to a decrease in antithyroid drug treatment failures, faster remission of the disease, and improved quality of life. The GRASS and CATALYST trials are being performed by the same group of investigators and both expected to be completed in 2018.

#### 3.2.4. Selenium and Immune Function

The supplementation with selenium, even in individuals without deficit of this micronutrient, has significant immune stimulatory effects. In fact, there is an improvement in the proliferation of activated T cells, increased tumour cytotoxic lymphocyte-mediated toxicity, and increased natural killer (NK) cell activity [1].

**Figure 3:**
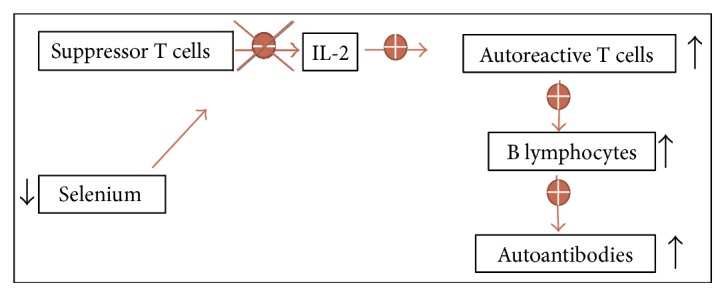
Selenium and immunity: when there is a selenium deficiency, suppressor T cells do not inhibit the production of some interleukins and this results in stimulation of autoreactive T cells, with the production of autoantibodies.

Studies performed in mice with selenium deficit showed that they had a reduced amount of mature and functional T cells, as well as failure of T cells to suppress the production of oxygen free radicals, with subsequent overproduction of oxidants followed by suppression of T cell proliferation [40]. Selenomethionine inhibits IFN-*γ*, TNF-*α*, and IL-2, and this effect is enhanced when combined with levothyroxine treatment (Figure 3). T cells are especially sensitive to oxidative stress, and T cells with deficit of selenoproteins cannot proliferate in response to the stimulation of their receptor, due to its inability to suppress the production of oxygen free radicals.

#### 3.2.5. Selenium and Cancer

Several studies evaluated the relationship between selenium levels in serum, plasma, and urine and cancer [41]. Overall, lower selenium levels have been associated with increased cancer diagnoses. Concerning thyroid pathology, Shen et al. [42] performed a meta-analysis comprising eight articles and 1291 subjects to clarify the association of selenium, copper, and magnesium levels with thyroid cancer. Overall, the authors concluded that patients with thyroid cancer had lower serum selenium and magnesium levels and higher copper levels when compared with healthy controls. Jonklaas et al. [43] performed a study with 65 euthyroid patients who were scheduled for thyroidectomy because of thyroid cancer, suspicion of thyroid cancer, or nodular disease. The results obtained suggest a potential association between lower selenium concentrations and higher thyroid cancer stage. Although the specific mechanisms are not yet fully understood, it seems that the antioxidant properties of selenoenzymes are relevant in carcinogenesis and tumour progression.

#### 3.2.6. Selenium, Overall Risk of Disease, and Mortality

Some trials show that there is a U-shaped relationship between selenium concentration in the blood and the risk of disease, with possible harm occurring both below and above the physiological range for optimal activity of some or all selenoproteins [44]. Therefore, supplementation should be recommended to patients with low levels of selenium. On the other hand, high selenium intake in individuals without proved deficit may have important adverse effects such as hyperglycaemia and atherosclerosis [45, 46].

Selenium levels correlate with mortality from all causes: there is an optimal range of concentration of this micronutrient, below and above which there appears to be increased mortality [1, 2]. In fact, a nonlinear association was noted between selenium status and all-cause and cancer mortality in a study with 13,887 participants with a follow-up of 12 years. In this study, at selenium levels greater than 150 ng/mL, there was a small positive association between serum selenium levels and all-cause and cancer mortality [47].

## 4. Conclusions

The maintenance of a physiological concentration of selenium (selenostasis) through a balanced diet or, alternatively, via supplementation is a prerequisite not only to prevent thyroid disease but also to maintain overall health. Selenium has a U-shaped relationship with disease, and either the deficiency or the excess of this micronutrient may be associated with adverse outcomes. In fact, there is a selenium concentration range in the body in which selenium benefits seem to be maximized.

Selenium supplementation in patients with Hashimoto's thyroiditis and reduced intake of this micronutrient may be useful, even for those who are already being treated with levothyroxine, although further studies are needed to confirm this benefit.

In patients with mild to moderate Graves' orbitopathy, selenium supplementation seems to be beneficial and the organic formula (selenomethionine) seems to be more advantageous than the inorganic formula.

## Figures and Tables

**Table 1:** Studies investigating selenium intake and concentration in water and food in Europe.

Country	Study	Subject number	Se intake/water and food content
UK [48]	Longitudinal study of healthy British adults using biochemical and molecular biomarkers	63	Women: 43 *μ*g/day Men: 54 *μ*g/day Average intake: 54 *μ*g/day

Spain [49]	Food intake and serum selenium concentration in elderly people	205	Women: 94.4 ± 23.6 *μ*g/day Men: 107.1 ± 32.2 *μ*g/day

France [50]	Case-control study of Se in people exposed to Se concentration in drinking water greater than the maximum recommended limit (10 *μ*g/L) using an FFQ	40 exposed subjects and 40 nonexposed controls	Exposed subjects' intake: 64 ± 14 *μ*g/day Nonexposed subjects' intake: 52 ± 14 *μ*g/day

Belgium [4]	To determine the Se status of the population	800 food products	Mean dietary Se intake: 60 *μ*g/day

Republic of Slovenia [51]	Cross-sectional study to assess Se status during 3 months of basic military training in a group of recruits using analysis of diet samples	15 recruits	48 ± 10 *μ*g/day

Italy [52]	Cross-sectional study of Se concentration in human milk after delivery compared to infant intake of Se from breast milk	242 women and their breastfeeding infants	Mean serum selenium concentration in milk: 12.1 ± 3.0 ng/g Mean selenium intake in infants: 9.5 ± 2.4 *μ*g/day

Northern Ireland [53]	Case-control study of chronic heart failure patients using a 4-day food diary	37	Selenium intake: 40.4–43.0 *μ*g/day

**Table 2:** Recommended dietary allowance of selenium for adults (*μ*g/day) [6, 7].

Country/region	Males	Females
Australia, 1990	85	70
Belgium, 2000	70	70
DACH (Germany, Austria, Switzerland), 2015	70	60
EC Scientific Committee on Food, 2003	55	55
France, 2001	60	50
Italy, 1996	50	40
Japan, 1999	55–60	45
New Zealand and Australia (proposed levels)	65	55
Nordic countries, 1996	50	40
USA and Canada, 2000	55	55
UK (Committee on Medical Aspects of Food Policy), 1991	75	60
World Health Organization/Food and Agriculture Organization/International Atomic Energy Agency, 1996	40	30

**Table 3:** Main groups of selenoproteins found in the thyroid gland and their function [1, 2].

Glutathione peroxidase	GPX	Catalyzes the reduction of H_2_O_2_ and protects against oxidative stress

Cytosolic GPx 1	GPX1	Antioxidative defense
Gastrointestinal GPx 2	GPX2	Antiapoptotic function in colon crypts; helps to maintain intestinal mucosal integrity
Extracellular GPx 3	GPX3	Antioxidant in extracellular fluid; thyroid protection from hydrogen peroxide in thyrocytes and follicular lumen
Phospholipid GPx 4	GPX4	Reduces the phospholipids' hydroperoxides; regulates apoptosis

Iodothyronine deiodinase	DIO	Production of active thyroid hormone T3, reverse T3 (rT3), and T2

Type I DIO	DIO1	Conversion of T4 to T3
Type II DIO	DIO2	Local production (intracellular) of T3 from T4
Type III DIO	DIO3	Production of rT3 from T4 and T2 from T3

Thioredoxin reductase	TXNRD	Oxidoreductase activity having NADPH as a cofactor

TXNRD cytosolic	TXNRD1	Main antioxidant at the cellular level
TXNRD mitochondrial	TXNRD2	Regulates cell proliferation
